# Supplementary material for: Modeling and Phenotyping Acute and Chronic Type 2 Diabetes Mellitus In Vitro in Rodent Heart and Skeletal Muscle Cells
Source: Cells. 2023 Dec 7;12(24):2786. doi: 10.3390/cells12242786 (PMC10741513; doi:10.3390/cells12242786)
Supplement: Supplementary file 1 [file cells-12-02786-s001.zip › cells-2665230-supplementary.pdf]

Supplements

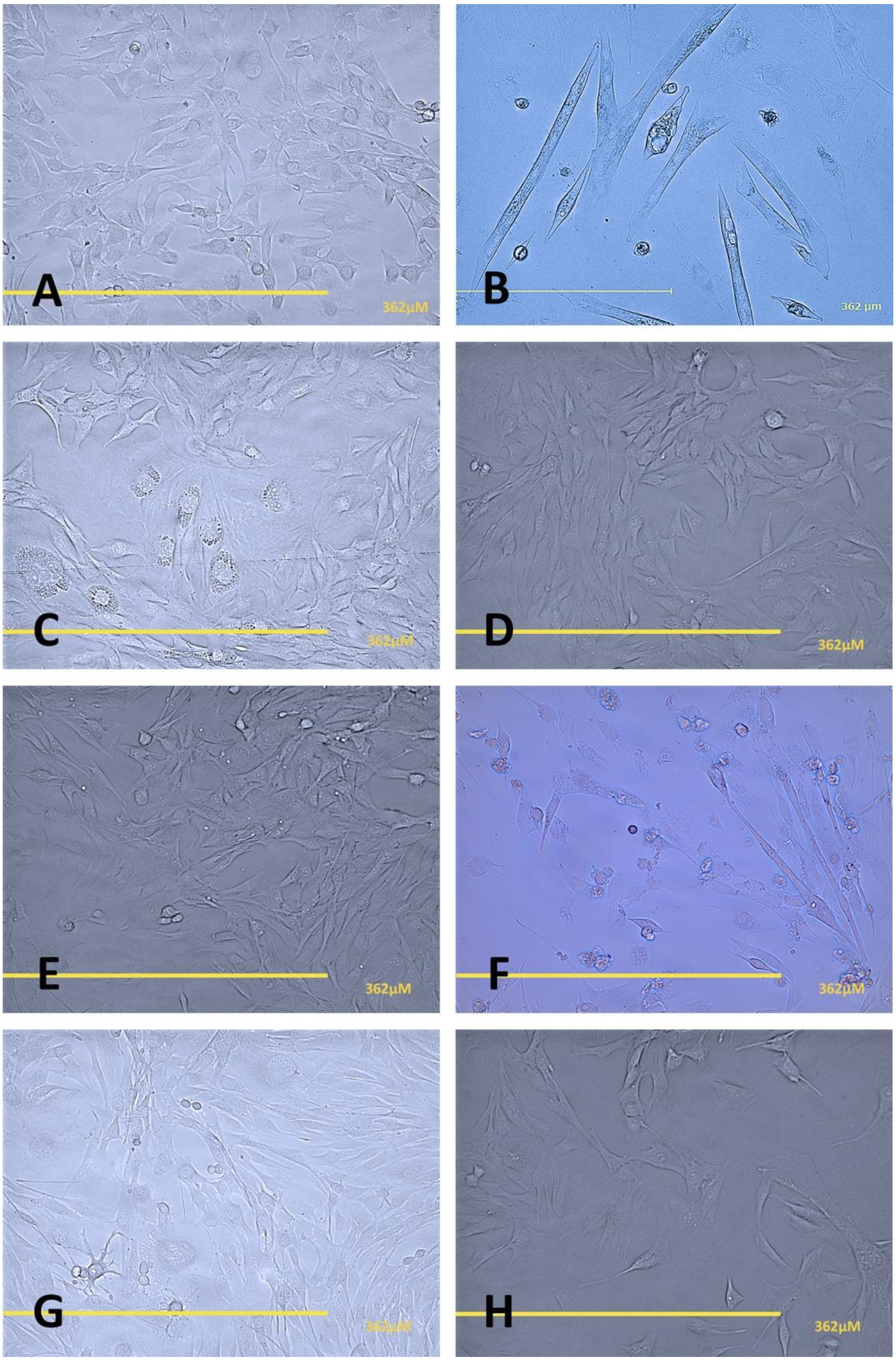

Fig. S1: C2C12 myoblasts and -tubes in different treatment conditions. A: Myoblasts in LG control growth medium. B: Myotubes in LG differentiation medium after 5 days of differentiation. C, D, E: Myoblasts after 96h HM (C), HG (D) and HGI (E) treatment. F: Myoblasts after 24h treatment in 750µM palmitate. G, H: Myoblasts after 96h LGP (G) and HGP (H) treatment, photographed at final concentrations of 150µM palmitate. Pictures were taken in a 20x magnification.

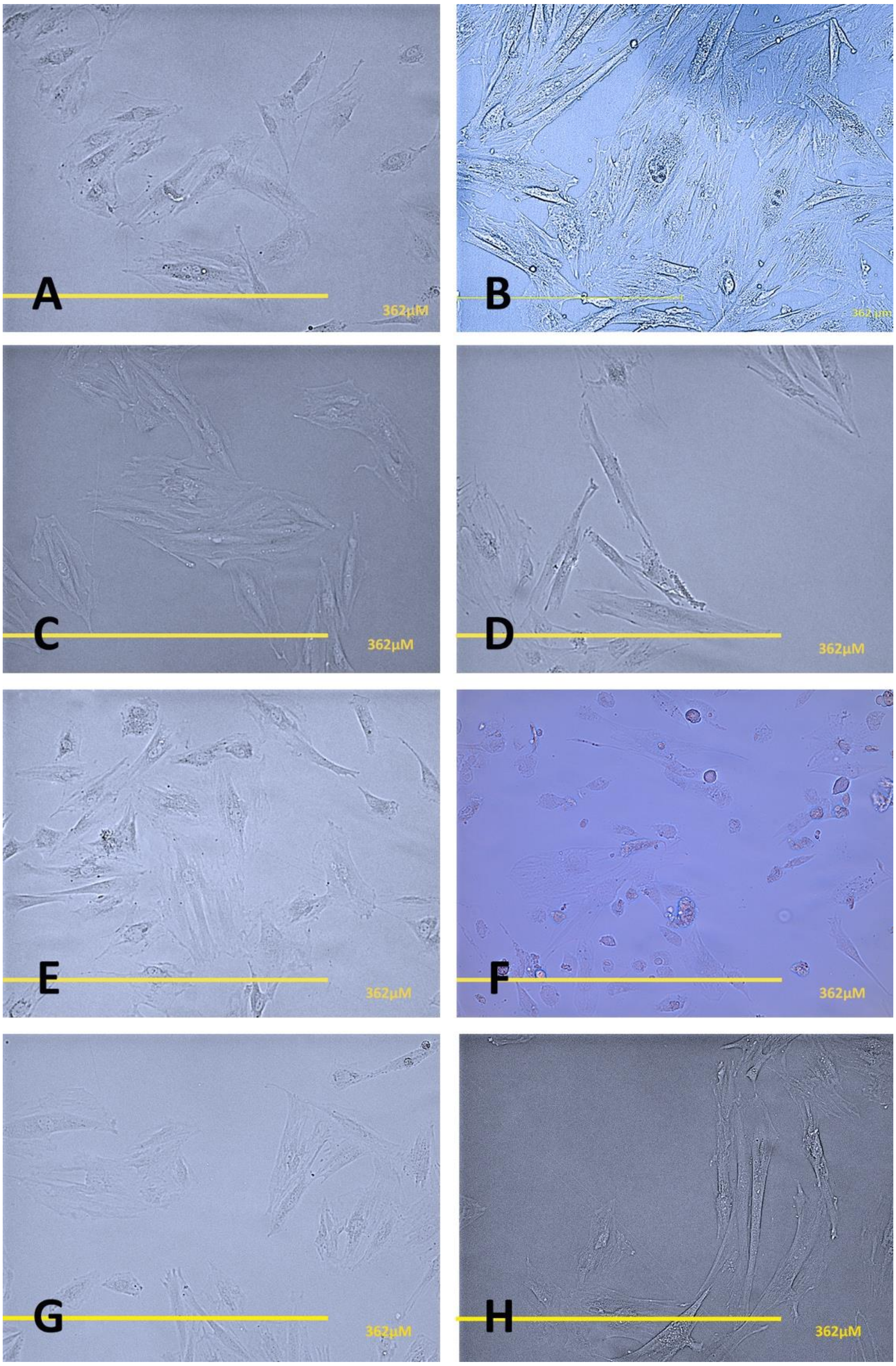

Fig. S2: H9C2 myoblasts and -tubes in different treatment conditions. A: Myoblasts in LG control growth medium. B: Myotubes in LG differentiation medium after 5 days of differentiation. C, D, E: Myoblasts after 96h HM (C), HG (D) and HGI (E) treatment. F: Myoblasts after 24h treatment in 750µM palmitate. G, H: Myoblasts after 96h LGP (G) and HGP (H) treatment, photographed at final concentrations of 150µM palmitate. Pictures were taken in a 20x magnification.

Table S1: Number of viable cells per visual field in increasing palmitate concentrations after 24 h incubation of C2C12 and H9C2 cells.

	LG	P 25 μM	P 50 μM	P 75 μM	P 100 μM	P 150μM	P 200μM	LGP 750μM
C2C12	66 ± 7	62 ± 3	65 ± 4	63 ± 5	62 ± 6	61 ± 3	25 ± 5	5 ± 1
H9C2	28 ± 5	29 ± 4	26 ± 6	26 ± 3	21 ± 1	17 ± 2	11 ± 3	7 ± 2
